# Latitudinal cline of ocean dependence in a diadromous fish

**DOI:** 10.1098/rspb.2024.2310

**Published:** 2025-01-29

**Authors:** Akihiko Goto, Mari Kuroki, Kentaro Morita

**Affiliations:** ^1^ Graduate School of Agricultural and Life Sciences, The University of Tokyo, 1-1-1 Yayoi, Bunkyo, Tokyo 113-8657, Japan; ^2^ Interfaculty Initiative in Information Studies, The University of Tokyo, 7-3-1 Hongo, Bunkyo, Tokyo 113-0033, Japan; ^3^ Atmosphere and Ocean Research Institute, The University of Tokyo, 5-1-5, Kashiwanoha, Kashiwa, Chiba 277-8564, Japan

**Keywords:** diadromy, anadromous migration, ocean dependence, food availability hypothesis, latitudinal cline, ocean-freshwater growth

## Abstract

Diadromous fishes exhibit latitudinal clines of ocean dependency at inter- and intra-species levels. A pattern of ocean dependence at high latitudes and river dependence at low latitudes is explained by relative aquatic productivity. Such latitudinal productivity clines may induce geographical variations in life-history diversity within migratory phenotypes. We hypothesized that the lifetime ocean dependency of a regional migratory salmonid would display a latitudinal cline that increased at higher latitudes. Freshwater growth rate decreased with higher latitudes, whereas marine growth rate was independent of latitude. The percentage of adult weight gain at sea was higher at higher latitudes. Relative weight gain (ln(ocean weight gain/freshwater weight gain)) decreased to zero at lower latitudes, indicating no growth benefit of going to sea at the southern distribution limit. These latitudinal variations in life history within salmonid migrants are consistent with the intra- and interspecific patterns and provide insight into the origin of diadromous migration but raise the question of whether the current definition of anadromy may be insufficient to fully capture the complexity and continuum of river-ocean migrations.

## 1. Introduction

**Figure 1.**
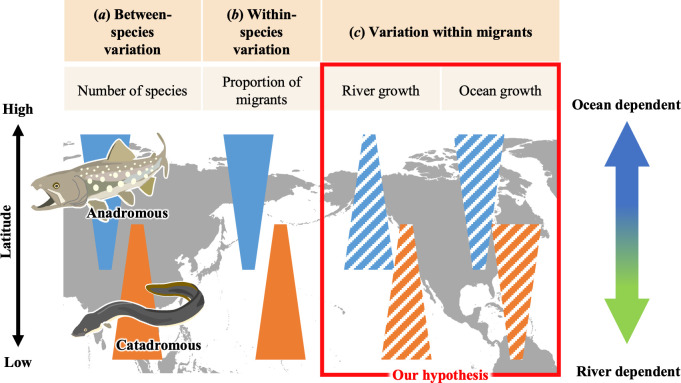
Schematic diagram of latitudinal variation in ocean-dependency-related traits of diadromous fish. (*a*) Between-species variation reviewed by McDowall [2], (*b*) within-species variation reviewed by Dodson *et al*. [3] and Tsukamoto *et al*. [4], and (*c*) our hypothetical variation within migrants. Blue, anadromous; orange, catadromous; stripes, our hypothesis.

Diadromous migration, by which fishes move between sea and freshwater habitats during their life cycles, has long captivated scientific inquiry. Diadromous migrants are classified into the following three types on the basis of differences in the use of oceans and rivers: anadromous fishes, which attain most of their growth in the sea and migrate to freshwater to breed; catadromous fishes, which attain most of their growth in freshwater and migrate to the sea to breed; and amphidromous fishes, the migration of which from freshwater to the sea, or *vice versa*, is not for the purpose of breeding [1]. The global geographic distribution of diadromous species has a latitudinal trend [2], with anadromous fishes being more common at high latitudes and catadromous fishes at low latitudes (figure 1*a*). Gross *et al*. [5] proposed a hypothesis relating these distribution patterns to global aquatic productivity. Their hypothesis suggested that divergent migratory behaviours could be attributed to the relative abundance of food in the ocean versus freshwater. In general, ocean productivity is higher at higher latitudes, producing more anadromous fishes, whereas freshwater productivity is higher at lower latitudes, producing more catadromous fishes [5].

Many diadromous fishes exhibit partial migration, whereby within the same population some individuals (called residents) remain in their natal habitats whereas others (called migrants) migrate to productive habitats [6]. Further research rooted in the food availability hypothesis has focused on latitudinal trends in the prevalence of dichotomous life histories (resident or migrant). Anadromous fish produce more migrants heading to the sea (hereafter, sea-run migrants) at higher latitudes [3,7–10], whereas catadromous fish produce more ocean residents at higher latitudes [4,11] (figure 1*b*). Collectively, the patterns of life-history variation within diadromous fish species indicate the increased utilization of marine environments at higher latitudes, a trend that is consistent with the patterns of variation observed across species.

Historically, research into anadromous migratory strategies has focused mainly on a dichotomous framework: to sea or not to sea—that has been the question. However, recently, there has been a surge in interest in the diversity of life histories within migrants [12]. Recent advances in technology, such as the tracking and reconstruction of migration ecology using otolith microchemistry [13], biotelemetry and archival tags [14], have revealed the detailed life histories of individuals at various spatio-temporal scales. For example, individuals spend different periods of time in their natal habitats before migration [15]; some juveniles exploit estuaries then return upstream to freshwater to overwinter before migrating to the ocean [16], whereas some individuals (in so-called ‘premature migration’) migrate early from the sea to freshwater, several months before spawning [17]. Furthermore, some individuals stop migrating after maturation and spawning and temporarily or permanently shift to resident-like habits [18,19].

**Figure 2.**
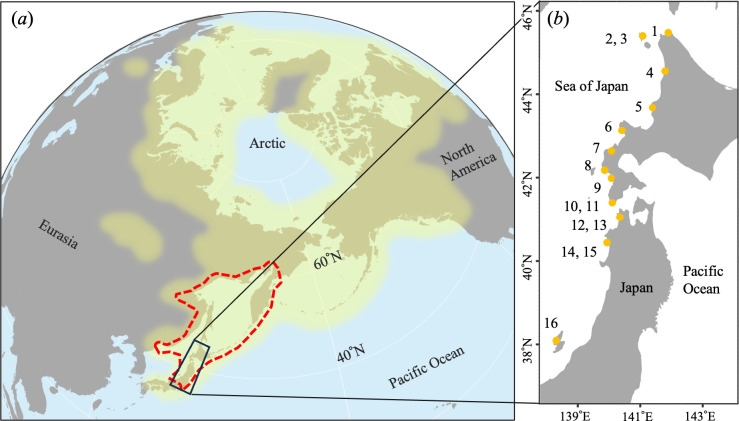
(*a*) Map of the distribution of genus *Salvelinus*. The yellow shading indicates the distribution of *Salvelinus* spp. and the red dashed line indicates the distribution of anadromous *Salvelinus leucomaenis*. The distributions were based on information provided by Taylor [21] and Esin and Markevich [22]. The black rectangle indicates our survey area. (*b*) Locations of the mouths of the surveyed rivers. The rivers’ names and latitudinal coordinates, and summarized fish sampling information, are shown in electronic supplementary material, table S1.

These spatio-temporal differences in ocean and river use in association with life-history diversity suggest that continuous variation in ocean dependency should occur within migrants. However, whether geographic variability in ocean dependency exists among conspecifics of the same migratory type remains unexplored. We hypothesize that the ocean dependency of sea-run migrants would increase at higher latitudes. Disparities in aquatic productivity are likely to influence the geographic trends of ocean dependency within migrants, yet empirical investigations of this subject are lacking. This issue can be clearly addressed by examining diadromous fish that display regional migration but have a widespread latitudinal distribution, such as charr, genus *Salvelinus* [20] (figure 2).

Our aim here was to test the following four hypotheses by using sea-run migrant white-spotted charr, *Salvelinus leucomaenis* (figure 1*c*): (i) the proportion of marine weight gain contributing to the total weight at breeding, i.e. ocean dependence, is higher at higher latitudes, (ii) growth rates during the freshwater life stage are lower at higher latitudes, (iii) growth rates during the marine life stage are higher at higher latitudes and (iv) relative weight gain (RWG) between pre- and post-migration is more advantageous in oceans at higher latitudes. If we were to find that the pattern of ocean dependency within migrants was consistent with the known intra- and interspecific patterns of ocean dependency [4,5,10], by which the ocean is utilized more at higher latitudes during the life history, then this finding would strongly support Gross’s hypothesis that the origin of diadromous migration is driven by relative productivity.

## 2. Methods

### (a) Study species

The white-spotted charr, *S. leucomaenis*, is an iteroparous anadromous fish with a multiannual spawning capacity and diverse migratory patterns. In this research, we focused on the typical anadromous life history, namely, that of a ‘normal annual migrant’, which accounts for over 80% of migrants [19]. Normal annual migrants spend several years in their freshwater natal rivers from birth until their initial seaward migration. Subsequently, the individuals that migrated to the ocean grew and returned to the river and repeated their annual migration, with upstream migration mainly for reproduction and seaward migration mainly for growth. The fish’s oceanic migration range is geographically narrow, typically within 10 km around the mouth of its natal river [23]. White-spotted charr are distributed from the Kamchatka Peninsula to northern Japan, corresponding to the southern limit of anadromous salmonids (figure 2*a*). Anadromous white-spotted charr comprise one genetic group with an isolation-by-distance population genetic structure [24].

### (b) Fish sampling

In August and September of 2019 and 2020, we collected white-spotted charr from 16 rivers (46°26′N to 38°06′N) flowing into the Sea of Japan (figure 2*b*; electronic supplementary material, table S1). The survey area was an optimal model region for verifying latitudinal clines; it exhibits a temperature gradient caused by the Tsushima Warm Current, with lower temperatures at higher latitudes (mean sea surface temperature: 15.4−21.6°C [25]; mean river temperature: 10.9−17.8°C [26], from May to September).

Sampling was conducted during the period when anadromous charr migrate upstream in rivers to spawn. Anadromous individuals with a fork length (FL) of 200 mm or more and lacking parr marks on their sides were captured using an electrofisher (Mod. 12B, Smith-Root Inc., WA, USA). Upon capture, FL (mm) and body weight (BW; g) were measured onsite. The gonads were dissected for visual sex determination and assessment of maturity. The sagittal otoliths were extracted from each individual, cleaned of surrounding tissue, and preserved in 99% ethanol for subsequent age determination and migration history analyses.

Individuals of the 117 sexually mature normal-annual anadromous white-spotted charr captured between 46°26′N and 38°06′N exhibited an average FL of 349.6 mm (range, 209–630 mm), an average age of 3.75 years (range, 2–8 years) and an average BW of 467.3 g (range, 95.6–2109 g). The estimated age at first sea entry averaged 2.58 years (range, 1–4 years), with FL at this time averaging 240.6 mm (range, 107.4–316.8 mm). The specimens were obtained from previous studies [19,27].

### (c) Otolith preparation

Otoliths were prepared in accordance with our previous protocol [27]. The distal side of the otoliths in 99% ethanol was photographed at ×1 magnification using a camera (DS-Fi1, Nikon, Japan) mounted on a stereomicroscope (SMZ1500, Nikon, Japan). The leading edge of the opaque zone that is formed annually from May to August [28] was considered to be the position of the annual rings (annuli). Using ImageJ (National Institute of Health, Bethesda, MD, USA), the length from the core to each annulus, as well as the otolith radius, was measured.

Following imaging, the otoliths were embedded in epoxy resin (EpoFix, Struers, Denmark) and mounted on glass slides for trace element analysis to identify migration patterns. The left otolith was used for the analysis unless it was not available, in which case, the right otolith was used. The otoliths were ground using silicon carbide papers (#800–#4000) on a polishing machine (RotoPol-35, Struers, Denmark) until the core was exposed, and they were then polished with OP-S suspension (Struers, Denmark). After being washed and dried, the otoliths were coated with platinum–palladium for 60 s using an ion sputter (E-1030, Hitachi, Japan). To analyse Sr and Ca, electron microprobe analyses were performed using an electron probe microanalyser (EPMA; JXA-8230, JEOL, Japan) at an acceleration voltage of 15 kV and a current of 12 nA. The measurement diameter was 9 μm, and the interval was 10 μm from core to edge along the same transect as used in the age analysis. To estimate the migration history, we used the value calculated as Sr:Ca×1000 (wt%/wt%) from the measured Sr and Ca (hereafter referred to as the Sr:Ca ratio).

### (d) Migration history reconstruction

We used changes in the Sr:Ca ratio along the otolith transect to identify the habitat shifts between the natal freshwater river and the ocean. We determined the first point exceeding the threshold value (4 × 10^3^) [29] as the otolith radius at the time of initial migration to the ocean (i.e. as smolt; O_smolt_), and the annulus closest to the O_smolt_ as indicative of the age of smoltification. We considered the increment from the core to the O_smolt_ as formed during the river period, and the increment beyond the O_smolt_ as formed during the ocean period. Salmonids are considered to grow exclusively in the ocean after their initial migration there [2,30]; therefore, any growth from the smolt stage to capture was assumed entirely as oceanic growth in subsequent growth analyses.

### (e) Growth analysis

We used otolith increments to estimate previous FLs and BWs. We estimated the FL at smoltification and at the time of formation of each annulus of each individual using the biological intercept method [31]:


(2.1) $$FL_{smolt}=FL_{capture}+\frac{\left(O_{smolt}-O_{capture}\right)\left(FL_{capture}-L_{0}\right)}{\left(O_{capture}-O_{0}\right)},$$

(2.2) $$FL_{t}=FL_{capture}+\frac{\left(O_{t}-O_{capture}\right)\left(FL_{capture}-L_{0}\right)}{\left(O_{capture}-O_{0}\right)}.$$

Here, *FL*_*smolt*_ represents the estimated FL at smoltification and *FL_t_* represents the estimated FL at age *t*. *FL*_*capture*_ is the FL at capture, *O*_*capture*_ is the otolith radius at capture, *O*_*smolt*_ is the otolith radius at smoltification, *O_t_* is the otolith annulus radius at age *t*, and *L*_0_ and *O*_0_ represent the length and otolith radius, respectively, of the emerged fry. We applied values recalculated from the work of Tsukamoto *et al*. [32] to *L*_0_ and *O*_0_ (*L*_0_ = 22.8 mm, *O*_0_ = 146 μm).

BWs at smoltification (BW_smolt_) and at age *t* (BW*_t_*) were estimated from the length–weight allometric relationship obtained from our dataset (BW = a*FL*^b^; a* = 0.0000136, *b* = 2.94, *n* = 117).

For the growth analysis, we used four indices, namely, marine per cent weight gain, annual growth rate, standardized growth rate and RWG. To examine ocean dependency as a proportion of the growth at capture, we calculated the marine per cent weight gain in accordance with the method of Quinn [33], namely, the increase in body weight in the ocean relative to the weight of the adult fish.

Annual growth rates were calculated using the difference between the estimated FL at age *t* and age *t*+1 for each individual. According to the reconstructed migration history, annual growth rates were categorized into riverine and oceanic growth rates. To remove the effect of initial size on annual growth rates, we also determined the size-standardized growth rates. These were determined as the residual from the linear regression of annual growth rate at age *t* on FL*_t_*.

To evaluate changes in growth associated with migration, we calculated the RWG using the following equation:


(2.3) $$RWG=ln\left(\frac{\Delta BW_{post-migration}}{\Delta BW_{pre-migration}}\right).$$

Here, Δ*BW*_*post-migration*_ is the ocean weight gain in the first migration year and Δ*BW*_*pre-migration*_ is the freshwater weight gain in the year just before seaward migration. The change in weight gain associated with migration is ocean advantageous at *RWG* > 0, river advantageous at *RWG* < 0 and balanced at *RWG* = 0.

### (f) Statistical analysis

To eliminate outliers in the regression between each growth index value and latitude, data with absolute values of studentized residuals exceeding three were not used in the analysis; one RWG value, one ocean growth rate value and two river growth rate values were omitted as outliers. The final dataset contained individual marine per cent weight gain (*n* = 117) and RWG (*n* = 116), as well as the annual growth rate and standardized growth rate in rivers (*n* = 300) and oceans (*n* = 252) for each age of the individual. Positive correlations were found between the annual growth rate and the standardized growth rate calculated for each age of each individual (river: *r* = 0.903, *n* = 300; ocean: *r* = 0.952, *n* = 252) and between the marine per cent weight gain and RWG calculated for each individual (*r* = 0.594, *n* = 116).

A linear mixed model (LMM) was employed to test the latitudinal trends in the objective variables. We constructed LMMs with annual growth rates in the ocean and rivers, standardized growth rates in the ocean and rivers, RWG and logit-transformed marine per cent weight gain (in accordance with the method of Warton and Hui [34]) as response variables. For the models of RWG or logit-transformed marine per cent weight gain, we included latitude (fixed effect, continuous), sex (fixed effect, categorical, male = 1, female = 0), the interaction term between latitude and sex (fixed effect), and river (random intercept effect) as predictor variables with a normal distribution of errors. Other basic life-history traits (FL and age at capture and smoltification) were similarly analysed using the LMMs. For the models of annual growth rate or standardized growth rate, individual (random intercept effect) was additionally included as a predictor variable along with the aforementioned variables. We performed *F*-tests using ANOVA for the LMMs to assess the significance of fixed effects. A *t*‐test was performed to test whether the mean RWG was significantly different from zero.

All statistical analyses were performed using R version 4.3.2 [35].

## 3. Results

### (a) Latitudinal trends in percentage adult weight gain at sea

**Figure 3.**
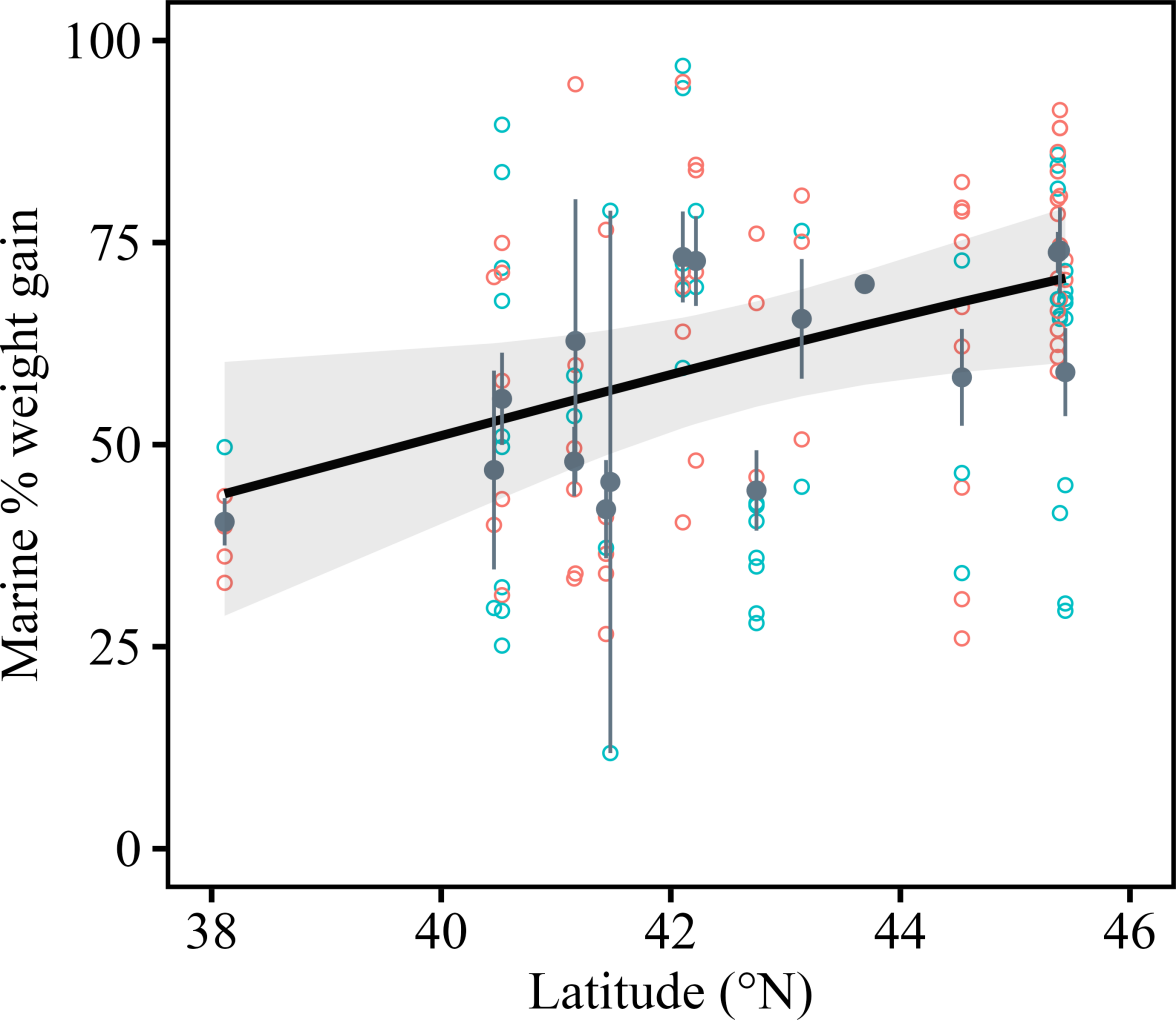
Latitudinal changes in marine % weight gain of sexually mature individuals. Mean (± s.e.), grey dots; males, blue dots; females, red dots. Shading shows the 95% CI of the fitted line predicted by latitude.

Although neither FL at maturity nor FL at first migration had a consistent latitudinal cline (electronic supplementary material, figure S1*a*,*c*; table S2), the percentage adult weight gain at sea (marine per cent weight gain) exhibited a clear pattern of being higher at higher latitudes and lower at lower latitudes (figure 3; table 1). The mean marine per cent weight gain across all individuals was 60%. In charr sampled from rivers at the lower latitude of 38°N, the marine per cent weight gain stood at 40%, whereas it reached approximately 70% in those sampled from rivers at higher latitudes, near 45°N. The age of sexually mature individuals and the age at first migration increased significantly with latitude (electronic supplementary material, figure S1*b*,*d*; table S2).

**Table 1.** Statistics of linear mixed models on ocean dependency and growth in anadromous white-spotted charr *Salvelinus leucomaenis*. The effects of latitude, sex (female = 0, male = 1) and the latitude×sex interaction term on marine % weight gain and growth parameters are detailed.

response variable	explanatory variable	coefficient	s.e.	statistics	***p-* value**
logit-transformed marine % weight gain	(intercept)	−7.883	3.10	—	—
	latitude	0.197	0.07	*F*_1, 12_ = 5.74	0.03
	sex	4.383	3.62	*F*_1, 112_ = 1.49	0.22
	latitude×sex	−0.107	0.08	*F*_1, 112_ = 1.57	0.21
annual river growth rate	(intercept)	267.513	53.49	—	—
	latitude	−4.428	1.25	*F*_1, 12_ = 21.10	0.001
	sex	109.697	53.53	*F*_1, 106_ = 0.28	0.60
	latitude×sex	−2.576	1.24	*F*_1, 109_ = 4.22	0.04
annual ocean growth rate	(intercept)	52.190	52.29	—	—
	latitude	0.047	1.22	*F*_1, 12_ = 0.02	0.89
	sex	20.268	51.12	*F*_1, 86_ = 1.20	0.28
	latitude×sex	−0.530	1.18	*F*_1, 93_ = 0.19	0.66
relative weight gain (RWG)	(intercept)	−3.183	1.37	—	—
	latitude	0.084	0.03	*F*_1, 12_ = 6.09	0.03
	sex	1.208	1.50	*F*_1, 110_ = 1.77	0.19
	latitude×sex	−0.030	0.03	*F*_1, 110_ = 0.74	0.39

### (b) Latitudinal trends in growth rate

**Figure 4.**
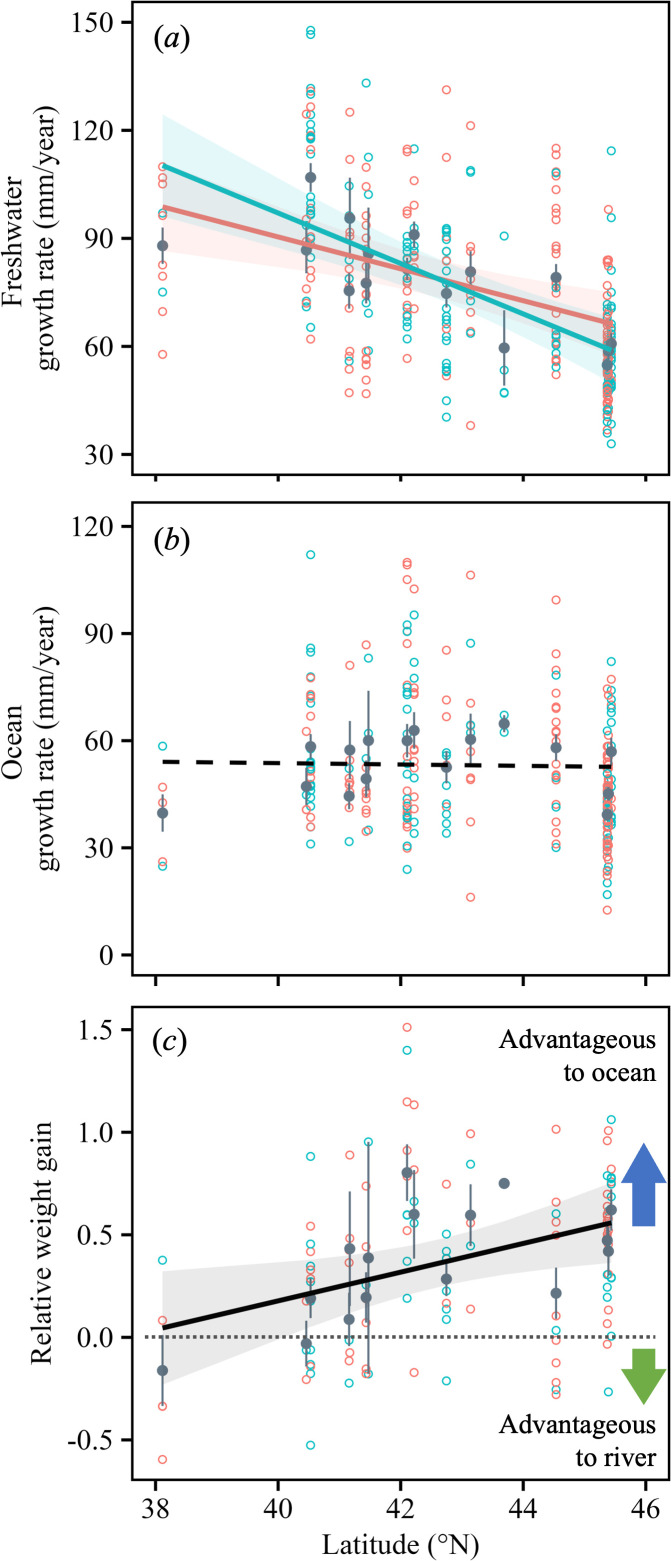
Latitudinal clines of growth rates and relative weight gain in rivers and oceans. Mean (± s.e.), grey dots; males, blue dots; females, red dots. Shadings show 95% CIs of the fitted lines predicted by latitude and the significant explanatory variables. The dashed line indicates no significant latitudinal cline. (*a*) Annual growth rate in rivers; (*b*) annual growth rate in oceans; (*c*) relative weight gain (RWG), namely, the weight gain in the year before migration as a ratio of that in the year after migration. The dotted line indicates the equilibrium point between growth in the ocean and growth in the river (*RWG* = 0).

The growth rate in freshwater (mm/year) decreased with higher latitude (figure 4*a*). Both the annual growth rate and the size-standardized growth rate in rivers were significantly influenced by latitude and sex, with males showing a steeper latitudinal cline than females (table 1, electronic supplementary material, figure S2*a* and table S3). Conversely, the growth rate in the ocean (mm/year) was independent of latitude (figure 4*b*). The annual growth rate and the size-standardized growth rate in the ocean did not increase significantly with latitude and did not differ between the sexes (table 1, electronic supplementary material, figure S2*b*; table S3).

### (c) Latitudinal trends in relative weight gain

The RWG, which we defined here as the ratio of weight gain during the year after the first seaward migration to that in the preceding year in the river, clearly increased with higher latitude (figure 4*c*). The RWG was significantly influenced by latitude but not by sex or by the interaction between latitude and sex (table 1). In 90 out of 116 individuals, the RWG was positive, indicating that there was a growth benefit of going to sea, and the mean RWG of 0.37 across all latitudes was significantly larger than zero (*t*‐test, *p* < 0.001). In contrast, the mean RWG was negative (−0.16) at 38°N, indicating that there was no growth benefit of going to sea at the southern limit of anadromy. Moreover, the 95% CI of the RWG overlapped with zero south of 40°N (figure 4*c*).

## 4. Discussion

**Figure 5.**
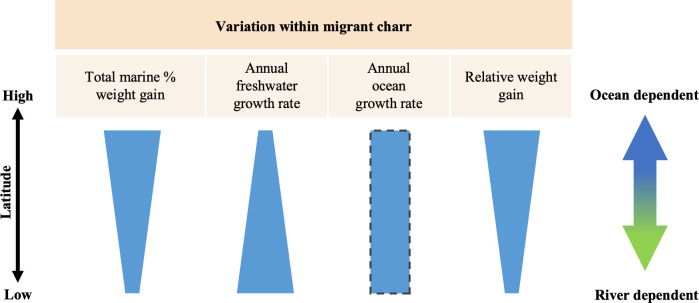
Latitudinal variations in ocean-dependency-related traits of anadromous *Salvelinus leucomaenis* based on the findings of this study. The rectangle with the dashed border indicates that there is no significant latitudinal cline.

Three out of four hypotheses regarding a latitudinal cline of migratory fish growth were supported. White-spotted charr, our study species, exhibited clear latitudinal trends in ocean dependence, river growth rate and RWG, supporting hypotheses (i), (ii) and (iv). But there was no discernible trend in ocean growth rate across latitudes, thus not supporting hypothesis (iii). In other words, migratory individuals at higher latitudes were more ocean dependent and those at lower latitudes were more river dependent (figure 5). This result is consistent with the empirical patterns shown by Gross *et al*. [5] that anadromy is prevalent at high latitudes and catadromy is common at low latitudes (i.e. diadromous fish are ocean dependent at high latitudes). Our study revealed that the food availability hypothesis can be valid within the same life-history form and promote life-history diversity.

### (a) Marine weight gain and life-history traits

A positive latitudinal trend was observed in the ocean dependency of weight gain at maturity. This implies that sea-run migrants depend highly on ocean growth at higher latitudes throughout their lifetimes at the individual level. Previous studies have examined latitudinal clines in various life-history traits, including size at maturity [36] and size at first migration [37], both of which were used to calculate marine per cent weight gain in our study (see §2). Whereas previous studies have discussed ocean dependency on the basis of various assumptions, our study achieved clear and accurate quantification at the individual level using otolith microchemical analysis techniques, specifically strontium to calcium (Sr:Ca) ratios. By utilizing the adult marine per cent weight gain as an indicator of ocean dependence, we successfully evaluated the proportion of ocean dependency in migrants at the individual level.

The unclear latitudinal trend in FL at initial migration can be explained by the size threshold for migration. Tsukamoto *et al*. [4] noted that diadromous fishes must first of all reach a certain age and size in order to start their migration. In other words, they must meet criteria for size-dependent locomotory ability, osmoregulatory capacity [38] and reduced mortality [39,40] in order to transit between ocean and freshwater habitats.

### (b) Relative growth potential between oceans and rivers

We found that the annual growth rate decreased at higher latitudes in rivers, whereas there was no significant latitudinal cline in the ocean growth rate. RWG, which is the ratio of annual weight gain achieved before and after migration to the sea, exhibited a positive trend with latitude. These differences in growth–latitude trends between the two habitats are highly suggestive, indicating that the potential for relative annual growth increases in the ocean at higher latitudes. This relative annual growth is responsible for generating a positive latitudinal trend in marine % weight gain, namely, the variation in ocean dependency along latitudes within migrants. Gross *et al*. [5] suggested that the contrasting migratory directions of diadromous fish are driven by latitudinal trends in the relative productivity of ocean and freshwater environments. The primary productivity in our study area also followed global trends, being higher at higher latitudes in the ocean and lower at higher latitudes on land [41,42]. Taking a step further, our study demonstrates that the migratory individuals of each population exhibit relative differences in growth potential along co-latitudinal trends in relative productivity.

In ectothermic organisms, growth rates are largely influenced by the complementary effects of food availability and environmental temperature [43]. In fishes [44], amphibians [45], crustaceans [46] and plants/algae [47,48], the annual growth decreases with decreasing water temperature, exhibiting a pattern of decrease from south to north latitudes. Particularly in rivers, low water temperatures strongly influence growth rates. Morita & Nagasawa [49] proposed that the latitudinal variation in the riverine growth of masu salmon juveniles is largely attributable to changes in temperature. Additionally, many studies have demonstrated that diadromous fish have decreased growth rates at higher latitudes in rivers (e.g. juvenile anadromous or landlocked salmonids [6,7,50] and upstream-migrating eels [51,52]). The negative trend between the riverine growth rate and latitude, as found in these studies and ours, is therefore likely a common pattern.

The steeper negative latitudinal cline of river growth in males may be due to sex differences in life-history strategies, which are influenced by early growth. In salmonids with partial migration, individuals with better early growth tend to become resident [53]. At lower latitudes, both males and females become resident [54]. However, at higher latitudes, females are almost entirely migrants, although males can also become residents [8,55]. Therefore, in this study, because we focused on sea-run migrants, resident males with high growth at higher latitudes were excluded from the dataset, thereby strengthening the negative latitudinal cline.

Unlike in rivers, we found no latitudinal trend in the growth rate in the ocean, even though, as in rivers, the ocean water temperature decreased towards higher latitudes. This pattern has also been observed in anadromous brown trout during the first year at sea [37]. However, latitudinal trends in ocean growth rates are not consistent; both positive [56] and negative [57] trends exist, indicating that ocean growth may not be regulated solely by water temperature. Food quantity and availability affect the growth rate of marine organisms [58]. Linse *et al*. [59] demonstrated that the growth and body size of benthic invertebrates can vary according to the local food conditions in southern polar waters at constant temperatures. Gross *et al*. [5] showed that the global ocean productivity is higher at higher latitudes. Furthermore, in the North Pacific region near our study area, the zooplankton abundance and biomass were higher at northern latitudes [60]. The difference in latitude-dependent growth trends between rivers and oceans may be attributable to these differences in the strength of the complementary effects of factors limiting growth; that is, in rivers, lower water temperature and less food availability synergistically suppress growth, whereas in oceans, the negative effect of lower temperature might be offset by the effect of increased food availability at higher latitudes.

The RWG—the ratio of weight gain during the year before the first migration to that after the first migration—exceeded zero in most individuals. This is evidence that the migrants experienced better growth in the ocean after migration. Conversely, at low latitudes, the RWG tended to approach or fall below zero. This suggests that little or no absolute growth benefits were derived from migration. The anadromy and residence of salmonids are most commonly explained by a conditional strategy involving status-dependent selection [3,61]. In this strategy, individuals with lower status in rivers enhance their fitness through migration. Conversely, if fitness improvement through seaward migration is not expected, then migration is unlikely to occur. Our findings suggest that south of a certain latitude (i.e. 38°N), the relative growth benefits diminish (*RWG* < 0), aligning with the southern distribution limit of anadromous migrants. At lower latitudes, the reduced RWG implies that the advantages of seaward migration do not offset the costs, leading to the absence of migration. White-spotted charr occurs south of this point but exhibits complete resident behaviour. Therefore, the RWG may be one of the factors regulating the southern limit of anadromy.

### (c) Latitudinal cline of ocean dependence is likely even stronger

The latitudinal cline of ocean dependence revealed among sea-run migrants is likely to be even more pronounced. Our assumption was that the species grew only in the ocean after the first sea entry, based on the conservative argument that post-maturity salmonids show no or little growth in rivers [2]. However, this argument has been challenged by our recent findings. We have reported [27] that more sea-run migrants river feed in the southern part than in the northern part of the distribution. Furthermore, in the southern distributions, we have observed retired migrant individuals that remain in the rivers after ascending from the ocean to spawn, predominating approximately half of the population [19]. However, this study used only normal annual migrants; retired migrants were not included in the results of the present analysis. These long-term stays and feeding in rivers, which occurred more frequently in the southern part of the distribution, should not be ignored when assessing the whole picture. Therefore, when considering these regional differences, it becomes evident that there is a much stronger latitudinal cline, with a river-dependent migratory strategy in the south and an ocean-dependent strategy in the north. Previous studies have overlooked geographical trends owing to their conservative assumptions. Future research that considers these factors may reveal additional trends in migratory diversity.

### (d) Will climate change reduce the ocean dependence of salmonids?

Geographical variations in ocean dependence among salmonid migrants can help predict how climate change will affect anadromous species. Salmonid fishes, which are considered to be among the optimal model organisms for predicting the impacts of global warming, could experience reduced distribution, degraded life-history diversity and compromised population viability [15,62].

According to the Intergovernmental Panel on Climate Change, global temperatures could rise by 2.7–4.4°C by 2100 [63], leading to a projected 5.4–11.6% decrease in ocean dependence for anadromous charr (see electronic supplementary material, Supplementary Information 5 figure S3). This would also shift the equilibrium latitude (*RWG* = 0) between growth advantages in the river and the ocean before and after the first migration northward to 39.5–41°N, implying a loss of anadromy for populations at the southern limit of their current distribution. As a result, cross-ecosystem linkages between rivers and oceans may degrade in the southern part of their range.

### (e) Questioning the definition of diadromy: anadromous fish with little marine growth

Our findings focusing on geographic variability in ocean dependency among conspecifics of the same migratory type raise the question of whether all migratory individuals can be considered ‘anadromous’. The marine per cent weight gain was lower in the south and was only 40% at the southern limit of the charr’s distribution. Even accounting for the potential underestimation of marine per cent weight gain (see electronic supplementary material, Supplementary Information 4), we can conclude that ocean dependence in the southernmost region is notably low. When compared with other salmonid species, which typically demonstrate marine per cent weight gains close to 99% [33], the gain of these charr remains notably lower. Although these charr generally fit the widely accepted definition of anadromous, they contradict it in several regards. Myers [1], a proponent of the term ‘diadromous’, defined ‘anadromous’ as follows: ‘Anadromous: diadromous fishes which spend most of their lives in the sea and migrate to fresh water to breed’. McDowall [2] noted that the key point of anadromy is a return migration to freshwater for breeding by fully grown, mature-to-ripe adults. However, our result of low marine per cent weight gain indicated that at least the aspect of ‘spending most of their lives in the sea’ may not qualitatively apply. Furthermore, instances of upstream migration—even at immature stages [64,65]—and feeding in rivers [27,66] make it hard to categorize these fish uniformly. Diadromous migration might be seen as a continuum, rather than as an anadromous/amphidromous/catadromous trichotomy, even within species.

## 5. Conclusion

We revealed clear latitudinal clines in ocean dependency among anadromous migrant charr *Salvelinus leucomaenis* by examining their ocean and river growth across a broad latitudinal range. While the food availability hypothesis—which posits that the origins of diadromous migration are driven by relative productivity—has previously been validated only at inter- and intra-species levels, our study is the first to demonstrate that this pattern holds true within individual migrants. Specifically, we confirm that diadromous fish are more dependent on oceans at higher latitudes and more dependent on rivers at lower latitudes, a pattern consistent across individual migrants, populations and species comparisons. Further research considering migration diversity as a continuum will provide deeper insights into the evolutionary mechanisms underlying migratory behaviour.

## Data Availability

Data used in this study are archived in the Dryad data repository [67]. Supplementary material is available online [68].

## References

[1] Myers GS. 1949 Usage of anadromous, catadromous and allied terms for migratory fishes. Copeia **2**, 89–97. (10.2307/1438482)

[2] McDowall RM. 1988 Diadromy in fishes: migrations between freshwater and marine environments. Portland, OR: Timber Press.

[3] Dodson JJ, Aubin‐Horth N, Thériault V, Páez DJ. 2013 The evolutionary ecology of alternative migratory tactics in salmonid fishes. Biol. Rev. **88**, 602–625. (10.1111/brv.12019) 23347290

[4] Tsukamoto K, Miller MJ, Kotake A, Aoyama J, Uchida K. 2009 The origin of fish migration: the random escapement hypothesis. Am Fish Soc Symp **69**, 45–61.

[5] Gross MR, Coleman RM, McDowall RM. 1988 Aquatic productivity and the evolution of diadromous fish migration. Science **239**, 1291–1293. (10.1126/science.239.4845.1291) 17833216

[6] Jonsson B, Jonsson N. 1993 Partial migration: niche shift versus sexual maturation in fishes. Rev. Fish Biol. Fish. **3**, 348–365. (10.1007/bf00043384)

[7] Armstrong RH, Morrow JE. 1980 The Dolly Varden charr, *Salvelinus malma*. In Charrs: salmonid fishes of the genus Salvelinus (ed. EK Balon), pp. 99–140. The Hague, The Netherlands: Dr. W. Junk.

[8] Yamamoto S, Morita K, Goto A. 1999 Geographic variations in life-history characteristics of white-spotted charr (Salvelinus leucomaenis). Can. J. Zool. **77**, 871–878. (10.1139/cjz-77-6-871)

[9] Antunes A, Faria R, Johnson WE, Guyomard R, Alexandrino P. 2006 Life on the edge: the long-term persistence and contrasting spatial genetic structure of distinct brown trout life histories at their ecological limits. J. Hered. **97**, 193–205. (10.1093/jhered/esj014) 16489148

[10] Maekawa K, Nakano S. 2002 To sea or not to sea: a brief review on salmon migration evolution. Fish. Sci. **68**, 27–32. (10.2331/fishsci.68.sup1_27)

[11] Rohtla M *et al*. 2023 Habitat use and growth of yellow-stage European eel in coastal and freshwater ecosystems in Norway. Can. J. Fish. Aquat. Sci. **80**, 14–26. (10.1139/cjfas-2022-0033)

[12] Quinn TP. 2021 Differential migration in Pacific salmon and trout: patterns and hypotheses. Anim. Migr. **8**, 1–18. (10.1515/ami-2021-0001)

[13] Campana SE. 1999 Chemistry and composition of fish otoliths: pathways, mechanisms and applications. Mar. Ecol. Prog. Ser. **188**, 263–297. (10.3354/meps188263)

[14] Cooke SJ, Hinch SG, Wikelski M, Andrews RD, Kuchel LJ, Wolcott TG, Butler PJ. 2004 Biotelemetry: a mechanistic approach to ecology. Trends Ecol. Evol. **19**, 334–343. (10.1016/j.tree.2004.04.003) 16701280

[15] Cordoleani F, Phillis CC, Sturrock AM, FitzGerald AM, Malkassian A, Whitman GE, Weber PK, Johnson RC. 2021 Threatened salmon rely on a rare life history strategy in a warming landscape. Nat. Clim. Chang. **11**, 982–988. (10.1038/s41558-021-01186-4)

[16] Koski KV. 2009 The fate of coho salmon nomads: the story of an estuarine-rearing strategy promoting resilience. Ecol. Soc. **14**, 4. (10.5751/es-02625-140104)

[17] Quinn TP, McGinnity P, Reed TE. 2016 The paradox of “premature migration” by adult anadromous salmonid fishes: patterns and hypotheses. Can. J. Fish. Aquat. Sci. **73**, 1015–1030. (10.1139/cjfas-2015-0345)

[18] Bond MH, Miller JA, Quinn TP. 2015 Beyond dichotomous life histories in partially migrating populations: cessation of anadromy in a long‐lived fish. Ecology **96**, 1899–1910. (10.1890/14-1551.1) 26378312

[19] Goto A, Kuroki M, Shirai K, Morita K. 2024 Diverse migration patterns of anadromous white-spotted charr Salvelinus leucomaenis revealed from otolith microchemistry. Ichthyol. Res. **71**, 508–521. (10.1007/s10228-023-00943-z)

[20] Balon EK (ed). 1980 Charrs: salmonid fishes of the genus Salvelinus. The Hague, The Netherlands: Dr W. Junk.

[21] Taylor EB. 2016 The Arctic char (Salvelinus alpinus) “complex” in North America revisited. Hydrobiologia **783**, 283–293. (10.1007/s10750-015-2613-6)

[22] Esin E, Markevich G. 2017 Charrs of genus Salvelinus of Asian North Pacific: origin, evolution and modern diversity. Petropavlovsk-Kamchatsky, Russia: Kamchatpress.

[23] Takami T. 1995 Migration of anadromous white-spotted charr, Salvelinus leucomaenis, in southwestern Hokkaido, Japan. Nord. J. Freshw. Res. **71**, 432–437.

[24] Yamamoto S, Morita K, Kitano S, Tabata R, Watanabe K, Maekawa K. 2023 Phylogeography of a salmonid fish, white-spotted charr (Salvelinus leucomaenis), in a historically non-glaciated region in the northwestern North Pacific. Biol. J. Linn. Soc. **139**, 115–130. (10.1093/biolinnean/blad002)

[25] Japan Meteorological Agency. 2024 Sea surface temperature information for coastal areas of Japan. See https://www.data.jma.go.jp/ kaiyou/data/db/kaikyo/series/engan/engan.html.

[26] Morita K, Tamate T, Kuroki M, Nagasawa T. 2014 Temperature‐dependent variation in alternative migratory tactics and its implications for fitness and population dynamics in a salmonid fish. J. Anim. Ecol. **83**, 1268–1278. (10.1111/1365-2656.12240) 24773465

[27] Goto A, Kuroki M, Morita K. 2023 Active feeding of anadromous white-spotted char Salvelinus leucomaenis at the southern latitudinal rivers. Can. J. Fish. Aquat. Sci. **80**, 1737–1747. (10.1139/cjfas-2023-0028)

[28] Yamamoto S, Nakano S, Tokuda Y. 1992 Variation and divergence of the life-history of Japanese charr Salvelinus leucomaenis in an artificial lake-inlet stream system. Jpn. J. Ecol. **42**, 149–157.

[29] Arai T, Morita K. 2005 Evidence of multiple migrations between freshwater and marine habitats of Salvelinus leucomaenis. J. Fish Biol. **66**, 888–895. (10.1111/j.0022-1112.2005.00654.x)

[30] Rutter C. 1903 Natural history of the Quinnat salmon: a report on investigations in the Sacramento River, 1896-1901. Bull. U.S. Fish. Comm. **22**, 65–141.

[31] Campana SE. 1990 How reliable are growth back-calculations based on otoliths? Can. J. Fish. Aquat. Sci. **47**, 2219–2227. (10.1139/f90-246)

[32] Tsukamoto K, Seki Y, Oba T, Oya M, Iwahashi M. 1989 Application of otolith to migration study of salmonids. Physiol. Ecol. Jpn. **Spec. 1**, 119–140.

[33] Quinn TP. 2018 The behavior and ecology of Pacific salmon and trout. Seattle, WA: University of Washington Press. (10.59962/9780774854610)

[34] Warton DI, Hui FKC. 2011 The arcsine is asinine: the analysis of proportions in ecology. Ecology **92**, 3–10. (10.1890/10-0340.1) 21560670

[35] R Core Team. 2023 R: A language and environment for statistical computing. R Found Stat Comput. See https://www.r-project.org/.

[36] Maekawa K, Nakano S. 2002 Latitudinal trends in adult body size of Dolly Varden, with special reference to the food availability hypothesis. Popul. Ecol. **44**, 17–22. (10.1007/s101440200002)

[37] Jonsson B, L’Abée‐Lund JH. 1993 Latitudinal clines in life‐history variables of anadromous brown trout in Europe. J. Fish Biol. **43**, 1–16. (10.1111/j.1095-8649.1993.tb01175.x)

[38] Varsamos S, Nebel C, Charmantier G. 2005 Ontogeny of osmoregulation in postembryonic fish: a review. Comp. Biochem. Physiol. Part A **141**, 401–429. (10.1016/j.cbpb.2005.01.013) 16140237

[39] Zabel RW, Williams JG. 2002 Selective mortality in chinook salmon: what is the role of human disturbance? Ecol. Appl. **12**, 173–183. (10.1890/1051-0761(2002)012[0173:smicsw]2.0.co;2)

[40] Futamura R, Morita K, Kanno Y, Kishida O. 2022 Size-selective mortality occurs in smolts during a seaward migration, but not in river residents, in masu salmon (Oncorhynchus masou). Environ. Biol. Fishes **105**, 1833–1843. (10.1007/s10641-022-01213-z)

[41] Ishizaka J, Yamada K. 2019 Phytoplankton and primary production in the Japan Sea. In Remote sensing of the Asian seas, pp. 177–189. Cham, Switzerland: Springer International Publishing. (10.1007/978-3-319-94067-0_9)

[42] Sasai T *et al*. 2011 Satellite-driven estimation of terrestrial carbon flux over Far East Asia with 1-km grid resolution. Remote Sens. Environ. **115**, 1758–1771. (10.1016/j.rse.2011.03.007)

[43] Angilletta MJ, Cooper BS, Schuler MS, Boyles JG. 2010 The evolution of thermal physiology in endotherms. Front. Biosci. **E2**, 861–881. (10.2741/e148) 20515760

[44] Heibo E, Magnhagen C, Vøllestad LA. 2005 Latitudinal variation in life-history traits in Eurasian perch. Ecology **86**, 3377–3386. (10.1890/04-1620)

[45] Morrison C, Hero J. 2003 Geographic variation in life‐history characteristics of amphibians: a review. J. Anim. Ecol. **72**, 270–279. (10.1046/j.1365-2656.2003.00696.x)

[46] Raper JLD, Schneider D. 2013 Spatial scaling from latitudinal gradients: growth rates in the American lobster Homarus americanus. Mar. Ecol. Prog. Ser. **483**, 231–243. (10.3354/meps10282)

[47] Rinde E, Sjøtun K. 2005 Demographic variation in the kelp Laminaria hyperborea along a latitudinal gradient. Mar. Biol. **146**, 1051–1062. (10.1007/s00227-004-1513-5)

[48] Kirwan ML, Guntenspergen GR, Morris JT. 2009 Latitudinal trends in Spartina alterniflora productivity and the response of coastal marshes to global change. Glob. Chang. Biol. **15**, 1982–1989. (10.1111/j.1365-2486.2008.01834.x)

[49] Morita K, Nagasawa T. 2010 Latitudinal variation in the growth and maturation of masu salmon (Oncorhynchus masou) parr. Can. J. Fish. Aquat. Sci. **67**, 955–965. (10.1139/f10-028)

[50] Jensen AJ, Forseth T, Johnsen BO. 2000 Latitudinal variation in growth of young brown trout Salmo trutta. J. Anim. Ecol. **69**, 1010–1020. (10.1046/j.1365-2656.2000.00457.x)

[51] Vélez-Espino LA, Koops MA. 2010 A synthesis of the ecological processes influencing variation in life history and movement patterns of American eel: towards a global assessment. Rev. Fish Biol. Fish. **20**, 163–186. (10.1007/s11160-009-9127-0)

[52] Jessop BM. 2010 Geographic effects on American eel (Anguilla rostrata) life history characteristics and strategies. Can. J. Fish. Aquat. Sci. **67**, 326–346. (10.1139/f09-189)

[53] Chapman BB, Hulthén K, Brodersen J, Nilsson PA, Skov C, Hansson L‐A, Brönmark C. 2012 Partial migration in fishes: causes and consequences. J. Fish Biol. **81**, 456–478. (10.1111/j.1095-8649.2012.03342.x) 22803720

[54] Kawanabe H. 1989 Japanese charrs and masu-salmon problems: a review. Physiol. Ecol. Jpn. **Spec. 1**, 13–24.

[55] Yamamoto S, Nakano S. 1996 Growth and development of a bimodal length‐frequency distribution during smolting in a wild population of white‐spotted charr in northern Japan. J. Fish Biol. **48**, 68–79. (10.1111/j.1095-8649.1996.tb01419.x)

[56] Conover DO, Present TMC. 1990 Countergradient variation in growth rate: compensation for length of the growing season among Atlantic silversides from different latitudes. Oecologia **83**, 316–324. (10.1007/bf00317554) 28313001

[57] Trip EDL, Clements KD, Raubenheimer D, Choat JH. 2014 Temperature‐related variation in growth rate, size, maturation and life span in a marine herbivorous fish over a latitudinal gradient. J. Anim. Ecol. **83**, 866–875. (10.1111/1365-2656.12183) 24252150

[58] Le Pape O, Bonhommeau S. 2015 The food limitation hypothesis for juvenile marine fish. Fish Fish. **16**, 373–398. (10.1111/faf.12063)

[59] Linse K, Barnes DKA, Enderlein P. 2006 Body size and growth of benthic invertebrates along an Antarctic latitudinal gradient. Deep Sea Res. Part II **53**, 921–931. (10.1016/j.dsr2.2006.03.006)

[60] Yamaguchi A, Matsuno K, Abe Y, Arima D, Imai I. 2017 Latitudinal variations in the abundance, biomass, taxonomic composition and estimated production of epipelagic mesozooplankton along the 155°E longitude in the western North Pacific during spring. Prog. Oceanogr. **150**, 13–19. (10.1016/j.pocean.2015.04.011)

[61] Gross MR. 1996 Alternative reproductive strategies and tactics: diversity within sexes. Trends Ecol. Evol. **11**, 92–98. (10.1016/0169-5347(96)81050-0) 21237769

[62] Keleher CJ, Rahel FJ. 1996 Thermal limits to salmonid distributions in the Rocky Mountain region and potential habitat loss due to global warming: a geographic information system (GIS) approach. Trans. Am. Fish. Soc. **125**, 1–13. (10.1577/1548-8659(1996)1252.3.co;2)

[63] Intergovernmental Panel on Climate Change (IPCC). 2021 Climate change 2021 – the physical science basis. Cambridge, UK and New York, NY, USA: Cambridge University Press. (10.1017/9781009157896)

[64] Rikardsen AH, Thorpe JE, Dempson JB. 2004 Modelling the life‐history variation of Arctic charr. Ecol. Freshw. Fish **13**, 305–311. (10.1111/j.1600-0633.2004.00070.x)

[65] Birnie-Gauvin K, Thorstad EB, Aarestrup K. 2019 Overlooked aspects of the Salmo salar and Salmo trutta lifecycles. Rev. Fish Biol. Fish. **29**, 749–766. (10.1007/s11160-019-09575-x)

[66] Garner SR, Heath JW, Neff BD. 2009 Egg consumption in mature Pacific salmon (Oncorhynchus spp.). Can. J. Fish. Aquat. Sci. **66**, 1546–1553. (10.1139/f09-103)

[67] Goto A, Kuroki M, Morita K. 2024 Latitudinal cline of ocean dependence in a diadromous fish. Dryad Digital Repository. (10.5061/dryad.r7sqv9smz) PMC11775620 39876724

[68] Goto A, Kuroki M, Morita K. 2025 Supplementary material from: Latitudinal cline of ocean dependence in a diadromous fish. Figshare (10.6084/m9.figshare.c.7611344) PMC11775620 39876724

